# Gender differences in the association between body mass index and recent suicide attempts in Chinese patients with first-episode drug-naïve major depressive disorder

**DOI:** 10.1038/s41598-023-43166-y

**Published:** 2023-09-25

**Authors:** Junjun Liu, Zhe Li, Fengnan Jia, Hsinsung Yuan, Yue Zhou, Xingzhi Xia, Ruchang Yang, Yuxuan Wu, Xiaobin Zhang, Gang Ye, Xiangdong Du, Xiangyang Zhang

**Affiliations:** 1 Nanjing Meishan Hospital, Nanjing, 210041 People’s Republic of China; 2 Department of Psychiatry, Suzhou Guangji Hospital, The Affiliated Guangji Hospital of Soochow University, Suzhou, 215137 People’s Republic of China; 3 Medical College of Soochow University, Suzhou, 215137 People’s Republic of China; 4 Xuzhou Medical University, Xuzhou, 221004 People’s Republic of China; 5 CAS Key Laboratory of Mental Health, Institute of Psychology, Chinese Academy of Sciences, Beijing, 100000 People’s Republic of China

**Keywords:** Diseases, Risk factors

## Abstract

Controversial evidence exists on the relationship between body mass index (BMI) and suicide attempts (SA) in patients with major depressive disorder (MDD). This cross-sectional study aimed to explore the association between BMI and SA in first-episode drug-naïve (FEDN) MDD patients in China. The study was conducted from 2016 to 2018 in Taiyuan, China. Univariate and multivariate logistic regression analyzed the BMI–SA association, with subgroup analysis for gender. Threshold effects were examined using two-piecewise regression. In males, BMI was significantly associated with SA (OR = 0.84, 95% CI 0.74–0.94, *P* = 0.003) after full adjustment, but not in females (OR = 0.97, 95% CI 0.89–1.06, *P* = 0.541). The interaction with gender was significant (*P* for interaction < 0.05). Smoothing plots revealed an L-shaped BMI–SA relationship in both genders, with BMI inflection points at 27.3 kg/m^2^ in males and 21.4 kg/m^2^ in females. Below the inflection points, BMI is negatively associated with SA in males (OR = 0.75, 95% CI 0.66–0.86, *P* < 0.001) and females (OR = 0.48, 95% CI 0.32–0.72, *P* < 0.001). Above the inflection points, no association existed for both genders (all *P* > 0.05). Results showed an L-shaped nonlinear BMI–SA relationship in FEDN MDD patients but differing BMI inflection points between genders, thus contributing to effective prevention programs for suicide.

## Introduction

Major depressive disorder (MDD) is a common serious mental disorder characterized by persistent depressed mood, loss of interest and motivation, sleep disturbance, changes in appetite, feelings of worthlessness or guilt, and even recurrent suicidal thoughts and behaviors^1^. The World Health Organization (WHO) ranked MDD as the third leading contributor to global disease burden in 2017, with predictions that it will become second by 2030^2^. At its most severe, depression can lead to suicide, which caused over 700,000 deaths worldwide in 2019, representing approximately 1.3% of all deaths^3^. A previous study reported that 63% of suicide decedents had MDD at the time of death^4^, with a lifetime prevalence of suicide attempts (SA) in people with MDD as high as 23.7%^5^. Moreover, past SA strongly predicts completed suicides and is associated with poorer long-term outcomes among survivors^6^.

The causes of SA in patients with MDD are complex, involving numerous biological, psychological, social, and environmental factors^7^. The epidemiology of suicide in depressed patients varies widely, influenced by factors including earlier onset^8^, male gender^9^, depressive severity^10^, past suicide attempts (SA)^11^, drug or alcohol abuse or dependence^12^, physical illness^13^, and physical inactivity^14^. In addition, some objective indicators, such as body mass index (BMI), an indicator of an unhealthy lifestyle, nutrition, physical activity, an unbalanced intake of energy, and insulin resistance, have also been the focus of researchers^15^. Factors affecting BMI may also influence neurodevelopment and the neuroendocrine system, which can affect the risk of developing mental illness^16^. However, the relationship between BMI and adult SA in patients of different genders is still unclear, with controversial research results. Some studies found low BMI associated with increased suicide risk in men, while high BMI correlated with suicidal ideation in women^17–19^. One study showed increased BMI in women linked to depression and suicidal thoughts, while lower BMI in men tied to depression, attempts, and ideation^20^. Another study found that obesity was associated with a higher suicide risk in women but not in men^21^. Inconsistencies across studies may stem from differing ethnicities, populations, hospitalization histories, antidepressant use, or other factors, making it difficult to fully understand or compare the results.

First-episode drug-naïve (FEND) MDD patients were defined as having a first symptomatic episode but no prior antidepressant or other medication use^22^. Patients with FEND MDD provide a unique opportunity to examine the relationship between SA and BMI in different genders without lifestyle or pharmacotherapy confounders like disease course or medical comorbidities. However, to our knowledge, no studies have specifically examined the association between BMI and SA in patients with FEND MDD, especially in different sex groups. Therefore, this cross-sectional study aimed to assess the relationship between BMI and SA in a relatively large sample of FEND MDD patients in China, stratified by gender.

## Materials and methods

### Study population

The study protocol adhered to the Declaration of Helsinki and was approved by the ethics committee of the First Hospital of Shanxi Medical University (No. 2016-Y27), with all participants providing written informed consent. All research was performed in accordance with relevant guidelines and regulations.

**Figure 1:**
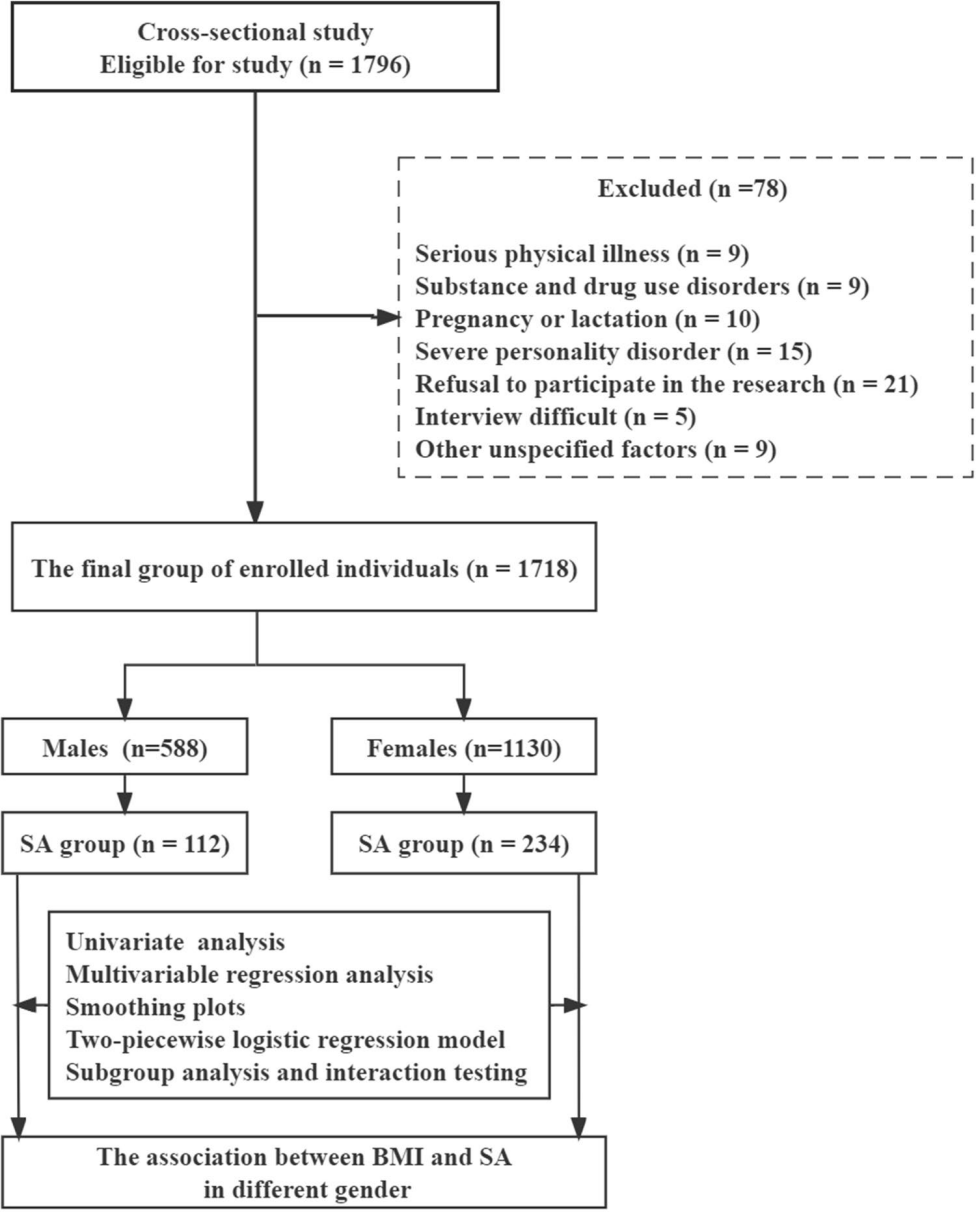
Flow chart of this study.

This cross-sectional study was conducted at the First Hospital of Shanxi Medical University (Taiyuan, Shanxi Province, China) from September 2016 to December 2018. Inclusion criteria were: (1) first-episode patients with MDD without medication; (2) age between 18 and 60 years; and (3) clinical diagnosis of MDD in accordance with the structured clinical interview of the Diagnostic and Statistical Manual of Mental Disorders, Fourth Edition (DSMIV). Exclusion criteria included the following: (1) serious physical illness such as cancer, persistent infection, epilepsy, brain injury, stroke, and diabetes (n = 9); (2) pregnant or breastfeeding (n = 10); (3) alcohol or drug abuse (n = 9); (4) severe personality disorder or other mental disorders that affect BMI, such as eating disorders (n = 15); (5) refusal to participate (n = 21); (7) unable to be interviewed due to an acute clinical condition (n = 5); and (8) other unknown reasons (n = 9) (Fig. 1). A total of 1796 patients were recruited and diagnosed by two experienced psychiatrists, with 78 excluded, leaving 1,718 participants (588 males and 1130 females).

### Socio-demographic characteristics and anthropometric data

Sociodemographic and clinical data were collected via a structured questionnaire, including age, sex, education, marital status, age of onset, and duration of illness. Body weight, height, systolic blood pressure (SBP), and diastolic blood pressure (DBP) were measured using standard calibrated protocols. Body mass index (BMI) was calculated as weight (kg) divided by height squared (m^2^).

Suicide attempt history was assessed through a face-to-face interview with the question “Have you ever attempted suicide in your lifetime?”, derived from the WHO/EURO multicenter study^23^. Patients with FEND MDD who responded “yes” to this inquiry were regarded as having attempted suicide. Then, we probed more into the frequency, mode, and precise dates of suicide attempts. Totally, 346 MDD patients were reported to have SA within the previous month. Of which, one patient attempted suicide four times, two patients three times, 26 patients twice, and 317 patients once. Participants were stratified into suicide attempt and non-attempt groups by gender based on their responses.

### Clinical assessment

Two qualified psychiatrists used the Chinese version of the Modified Structured Clinical Interview for DSM-IV-TR criteria (SCID-I/P) to provide a consensus diagnosis of MDD for each patient.

The 17-item Hamilton Depression (HAMD-17) Chinese version was used to assess depression severity. It contains 17 items, with 8 scored on a 5-point scale from 0 (absent) to 4 (severe) and 9 scored from 0 (absent) to 2 (symptom-specific severity). Total scores range from 0 to 52, with higher scores indicating more severe depression. The HAMD-17 has demonstrated strong validity and reliability in China and has been widely used in previous research^24^.

The Hamilton Anxiety Rating Scale (HAMA) Chinese version was used to evaluate anxiety symptom severity^25^. It consists of 14 symptom-defined items scored on a 5-point Likert scale from 0 (absence) to 4 (severe). Total scores range from 0 to 56, encompassing both somatic anxiety (e.g., physical discomfort) and psychogenic anxiety (e.g., mental agitation, psychological distress).

Two certified psychiatrists with over 5 years of clinical experience received pre-study training on the use of the rating scales. Inter-observer reliability for HAMA and HAMD total scores post-training remained above 0.8 with repeated assessments. Additionally, they were unaware of the patients’ clinical conditions.

### Blood samples

Blood samples were taken from each participant between the hours of 6:00 and 9:00 am after an overnight fast. On the same day, all samples were submitted to the hospital’s laboratory facility and measured before 11:00 a.m. Using an automated clinical ARCHITECT Immulite 2000 SR analyzer, the following hormones were measured: free triiodothyronine (FT3), free thyroxine (FT4), thyroid stimulating hormone (TSH), antithyroglobulin (TgAb), and thyroid peroxidase antibody (TPOAb) (Abbott, Longford, Ireland). An auto-analyzer was used to measure the levels of fasting blood sugar and the lipid profile, comprising low-density lipoprotein cholesterol (LDL-C), high-density lipoprotein cholesterol (HDL-C), cholesterol (TC), and triglyceride (TG) (ARCHITECT c8000 system, Abbott Laboratories, Illinois, USA).

### Statistical analysis

Continuous variables were assessed for normality using the Kolmogorov–Smirnov test. Normally distributed variables were summarized as mean ± standard deviation (SD), and non-normally distributed variables as median and interquartile range (IQR). Categorical variables were expressed as frequencies and percentages (%). Characteristic differences between male and female patients were examined using independent t-tests and Mann–Whitney U tests for continuous data and chi-square tests for categorical data. The linear and nonlinear relationships between SA and BMI were evaluated using multivariate linear regression models and two-piece piecewise regression models, adjusting for age at onset, length of illness, education, HAMA, HAMD, TSH, TGAb, TPOAb, FBG, TC, TG, HDL-c, TG, LDL-c, systolic pressure, and diastolic pressure in men and women. Stratified and interaction analyses were further conducted to explore the potential modifier and interaction effects on the SA-BMI association. Variables with a variance inflation factor (VIF) > 5 were excluded from the final model to avoid multicollinearity. Covariates were also screened and included if they significantly altered the BMI–SA estimate by > 10% or were associated with SA (*P* < 0.10)^26^. Log-likelihood ratio tests compared differences between regression models.

The statistical software programs R 4.3.0 (http://www.r-project.org, The R Foundation) and EmpowerStats (http://www.empowerstats.com, X&Y Solution, Inc., Boston, Massachusetts, USA) were used for all analyses. Statistical significance was defined as a two-tailed *p*-value < 0.05.

## Results

### Characteristics of the participants

A total of 1,718 FEDN MDD patients met the criteria for enrollment and were included in this study, with 588 males and 1130 females, ages 34.87 ± 12.43 years (range: 18–60 years), and a mean disease duration of 5.00 (3.00, 8.00) months. The overall mean level of BMI was (24.37 ± 1.92 kg/m^2^), (24.41 ± 2.04 kg/m^2^) among men, and (24.35 ± 1.86 kg/m^2^) among women. The prevalence of SA in male and female patients was 19.05% (112/588) and 20.71% (234/1130), respectively (Table 1).

**Table 1:** Comparison of socio-demographic and clinical characteristics between male and female patients with FEDN MDD.

Characteristics	Total (n = 1718)	Males (n = 588)	Females (n = 1130)	*P*-value
Age (years)	34.87 ± 12.43	33.12 ± 12.22	35.78 ± 12.45	< 0.001
Age at onset (years)	34.66 ± 12.31	32.91 ± 12.09	35.57 ± 12.34	< 0.001
Duration of illness (months)	5.00 (3.00, 8.00)	5.00 (3.00, 8.00)	5.00 (3.00, 8.00)	0.363
HAMD	30.30 ± 2.94	30.28 ± 2.95	30.30 ± 2.94	0.883
HAMA	20.80 ± 3.47	20.66 ± 3.38	20.87 ± 3.52	0.231
TSH (uIU/ml)	5.07 ± 2.56	4.95 ± 2.48	5.13 ± 2.60	0.157
TGAb (IU/l)	21.46 (14.43, 43.62)	21.19 (14.23, 37.30)	21.80 (14.46, 46.81)	0.355
TPOAb (IU/l)	17.43 (12.32, 34.61)	16.81 (12.48, 32.86)	18.20 (12.24, 35.27)	0.590
FT3 (pmol/l)	4.91 ± 0.72	4.95 ± 0.72	4.88 ± 0.72	0.055
FT4 (pmol/l)	16.70 ± 3.10	16.72 ± 3.15	16.69 ± 3.07	0.838
FBG (mmol/l)	5.40 ± 0.65	5.37 ± 0.64	5.41 ± 0.65	0.229
TC (mmol/l)	5.25 ± 1.11	5.22 ± 1.06	5.26 ± 1.13	0.429
HDL-c (mmol/l)	1.22 ± 0.29	1.22 ± 0.30	1.22 ± 0.28	0.629
TG (mmol/l)	2.17 ± 0.99	2.14 ± 1.00	2.18 ± 0.98	0.497
LDL-c (mmol/l)	2.98 ± 0.86	2.96 ± 0.85	3.00 ± 0.87	0.428
BMI (kg/m^2^)	24.37 ± 1.92	24.41 ± 2.04	24.35 ± 1.86	0.539
Systolic pressure (mmHg)	119.48 ± 10.91	118.76 ± 10.40	119.86 ± 11.16	0.047
Diastolic pressure (mmHg)	75.95 ± 6.74	75.90 ± 6.89	75.98 ± 6.67	0.820
Suicide attempts	346(20.14%)	112 (19.05%)	234 (20.71%)	0.416
Marital status				0.007
Single	502 (29.22%)	196 (33.33%)	306 (27.08%)	
Marriage	1216 (70.78%)	392 (66.67%)	824 (72.92%)	
Education				0.009
Junior high school	413 (24.04%)	125 (21.26%)	288 (25.49%)	
Senior high school	760 (44.24%)	271 (46.09%)	489 (43.27%)	
College	449 (26.14%)	170 (28.91%)	279 (24.69%)	
Postgraduate	96 (5.59%)	22 (3.74%)	74 (6.55%)	

FEDN, first-episode drug-naïve; MDD, major depressive disorder; HAMD, 17-item Hamilton Rating Scale for Depression; HAMA, 14-item Hamilton Anxiety Rating Scale; TSH, thyroid-stimulating hormone; TGAb, thyroglobulin antibody; TPOAb, thyroid peroxidase antibody; FT3, free triiodothyronine; FT4, free thyroxine; FBG, fasting blood glucose; TC, total cholesterol; HDL-c, high-density lipoprotein cholesterol; TG, triglyceride; LDL-c, low-density lipoprotein cholesterol; BMI, body mass index.

Female patients had higher levels of age, age at onset, systolic pressure, and were more likely to be married than male patients (all *P* < 0.05), but their duration of illness (months), HAMD, HAMA, TSH (uIU/ml), TGAb (IU/l), TPOAb (IU/l), FT3 (pmol/l), FT4 (pmol/l), FBG (mmol/l), TC (mmol/l), HDL-c (mmol/l), TG (mmol/l), LDL-c (mmol/l), BMI (kg/m^2^), and DBP (mmHg) were not significantly different (all *P* > 0.05). After Bonferroni corrections, there was no significant difference in education between different genders (*P* = 0.009, Bonferroni corrected *P* < 0.05/6 = 0.0083).

### Univariate logistic analysis of SA in males and females

The univariate logistic regression analysis was performed by taking SA as the dependent variable (Y = 1) and using the clinical data and blood indicators from different gender groups as independent variables (Table 2). The results showed that HAMD, HAMA, TSH (uIU/ml), TGAb (IU/l), TPOAb (IU/l), FBG (mmol/l), TC (mmol/l), HDL-c (mmol/l), LDL-c (mmol/l), BMI (kg/m2), systolic pressure (mmHg), and diastolic pressure (mmHg) were all significantly related to SA in male patients (*P* all < 0.05). In female patients, the association between duration of illness, HAMD, HAMA, TSH (uIU/ml), TGAb (IU/l), TPOAb (IU/l), FBG (mmol/l), TC (mmol/l), HDL-c (mmol/l), TG (mmol/l), LDL-c (mmol/l), systolic pressure (mmHg), diastolic pressure (mmHg), and SA was significant (all *P* < 0.05). Compared with junior high school patients, senior high school and college patients were negatively associated with SA (all *P* < 0.05).

**Table 2:** Univariate analysis for Suicide attempts in male and female patients with FEDN MDD.

Covariate	Males (n = 588)		Females (n = 1130)	
	OR (95% CI)	*P*-value	OR (95% CI)	*P*-value
Age (years)	1.01 (1.00, 1.03)	0.156	1.01 (1.00, 1.02)	0.139
Age at onset (years)	1.01 (1.00, 1.03)	0.161	1.01 (1.00, 1.02)	0.144
Duration of illness (months)	1.03 (0.99, 1.08)	0.103	1.03 (1.00, 1.06)	0.031
HAMD	1.31 (1.21, 1.41)	< 0.001	1.39 (1.31, 1.48)	< 0.001
HAMA	1.34 (1.25, 1.43)	< 0.001	1.37 (1.31, 1.44)	< 0.001
TSH (uIU/ml)	1.40 (1.27, 1.54)	< 0.001	1.38 (1.29, 1.46)	< 0.001
TGAb (IU/l)	1.00 (1.00, 1.00)	0.003	1.00 (1.00, 1.00)	< 0.001
TPOAb (IU/l)	1.00 (1.00, 1.00)	< 0.001	1.00 (1.00, 1.00)	< 0.001
FT3 (pmol/l)	0.84 (0.63, 1.11)	0.225	1.13 (0.92, 1.38)	0.233
FT4 (pmol/l)	1.01 (0.95, 1.08)	0.771	0.99 (0.94, 1.04)	0.672
FBG (mmol/l)	2.04 (1.48, 2.80)	< 0.001	1.60 (1.29, 1.99)	< 0.001
TC (mmol/l)	1.87 (1.52, 2.30)	< 0.001	1.67 (1.46, 1.91)	< 0.001
HDL-c (mmol/l)	0.29 (0.14, 0.60)	< 0.001	0.27 (0.16, 0.46)	< 0.001
TG (mmol/l)	1.12 (0.92, 1.36)	0.276	1.20 (1.04, 1.38)	0.013
LDL-c (mmol/l)	1.60 (1.25, 2.04)	< 0.001	1.39 (1.18, 1.64)	< 0.001
BMI (kg/m^2^)	0.89 (0.80, 0.99)	0.031	1.04 (0.96, 1.13)	0.299
Systolic pressure (mmHg)	1.04 (1.02, 1.07)	< 0.0001	1.06 (1.05, 1.08)	< 0.001
Diastolic pressure (mmHg)	1.05 (1.02, 1.08)	< 0.001	1.09 (1.06, 1.11)	< 0.001
Marital status				
Single	1.0 (reference)		1.0 (reference)	
Marriage	1.46 (0.93, 2.32)	0.104	0.96 (0.69, 1.32)	0.787
Education				
Junior high school	1.0 (reference)		1.0 (reference)	
Senior high school	0.93 (0.54, 1.60)	0.790	0.65 (0.46, 0.91)	0.014
College	1.01 (0.56, 1.82)	0.964	0.58 (0.39, 0.87)	0.009
Postgraduate	1.58 (0.56, 4.46)	0.389	0.90 (0.50, 1.62)	0.718

CI, confidence interval. OR, odds ratio.

### Linear relationship between SA and BMI in males and females

Multivariable logistic regression analysis was used to evaluate the linear association between BMI and SA by adjusting the covariates. The model with non-adjusted covariates equaled univariate logistic regression analysis. The minimally adjusted covariates (Adjust I) included age at onset, duration of illness, and education, and the fully adjusted covariates (Adjust II) included age at onset, duration of illness, education, HAMA, HAMD, TSH, TGAb, TPOAb, FBG, TC, TG, HDL-c, TG, LDL-c, systolic pressure, and diastolic pressure (Table 3). For male patients, an increase in BMI elevated the risk of SA in all three models (all *P* < 0.05), with an OR = 0.84 (95% CI 0.74–0.94, *P* = 0.003) in the fully-adjusted model. For female patients, the association between BMI and SA was not significant in all three models (all *P* > 0.05). The interaction effect between different genders was significant (all *P* for interaction < 0.05), indicating that the relationship between BMI and SA was affected by gender.

**Table 3:** Relationship between BMI and SA in different models.

Variable	n	Non-Adjusted		Adjusted I		Adjusted II	
		OR (95% CI)	*P*-value	OR (95% CI)	*P*-value	OR (95% CI)	*P*-value
Males							
BMI (kg/m^2^)	558	0.89 (0.80, 0.99)	0.031	0.88 (0.79, 0.98)	0.020	0.84 (0.74, 0.94)	0.003
BMI category							
< 18.5 kg/m^2^	3	6,846,260.28 (0.00, Inf)	0.975	7,748,200.55 (0.00, Inf)	0.975	8,879,381.13 (0.00, Inf)	0.984
18.5–23.9 kg/m^2^	237	1 (reference)		1 (reference)		1 (reference)	
24– 27.9 kg/m^2^	320	0.56 (0.36, 0.86)	0.008	0.53 (0.34, 0.82)	0.005	0.38 (0.22, 0.64)	< 0.001
≥ 28 kg/m^2^	28	0.88 (0.34, 2.28)	0.795	0.85 (0.32, 2.26)	0.744	0.44 (0.14, 1.37)	0.158
Females							
BMI (kg/m^2^)	1130	1.04 (0.96, 1.13)	0.299	1.04 (0.96, 1.12)	0.326	0.97 (0.89, 1.06)	0.541
BMI category							
< 18.5 kg/m^2^	7	5.90 (1.30, 26.88)	0.022	4.92 (1.06, 22.72)	0.041	7.85 (1.47, 41.84)	0.016
18.5–23.9 kg/m^2^	445	1 (reference)		1 (reference)		1 (reference)	
24–27.9 kg/m^2^	642	1.21 (0.89, 1.64)	0.216	1.18 (0.86, 1.60)	0.301	1.01 (0.70, 1.45)	0.963
≥ 28 kg/m^2^	36	1.70 (0.79, 3.67)	0.174	1.68 (0.77, 3.64)	0.190	1.26 (0.51, 3.11)	0.611
*P* for interaction		0.018		0.019		0.029	

BMI, body mass index; CI, confidence interval.

Model I adjusted for age at onset, duration of illness, education.

Model II adjusted for age at onset, duration of illness, education, HAMA, HAMD, TSH, TGAb, TPOAb, FBG, TC, TG, HDL-c, LDL-c, systolic pressure, diastolic pressure.

For sensitivity analysis, we converted BMI from a continuous variable to a categorical variable. Based on guidelines from the Working Group on Obesity in China (WGOC)^16^, participants were classified into BMI categories of underweight (< 18.5 kg/m^2^), normal weight (18.5–23.9 kg/m^2^), overweight (24–27.9 kg/m^2^), or obesity (≥ 28 kg/m^2^). Compared to those with normal weight, overweight males exhibited reduced odds of SA (OR = 0.38, 95% CI 0.22–0.64, *P* < 0.001), whereas underweight females showed increased odds of SA (OR = 7.85, 95% CI 1.47–41.84, *P* = 0.016). However, the small sample size of just 7 underweight females limits the statistical power; thus, the elevated SA risk associated with being underweight in females should be interpreted cautiously.

### Nonlinear relationship between SA and BMI in males and females

**Figure 2:**
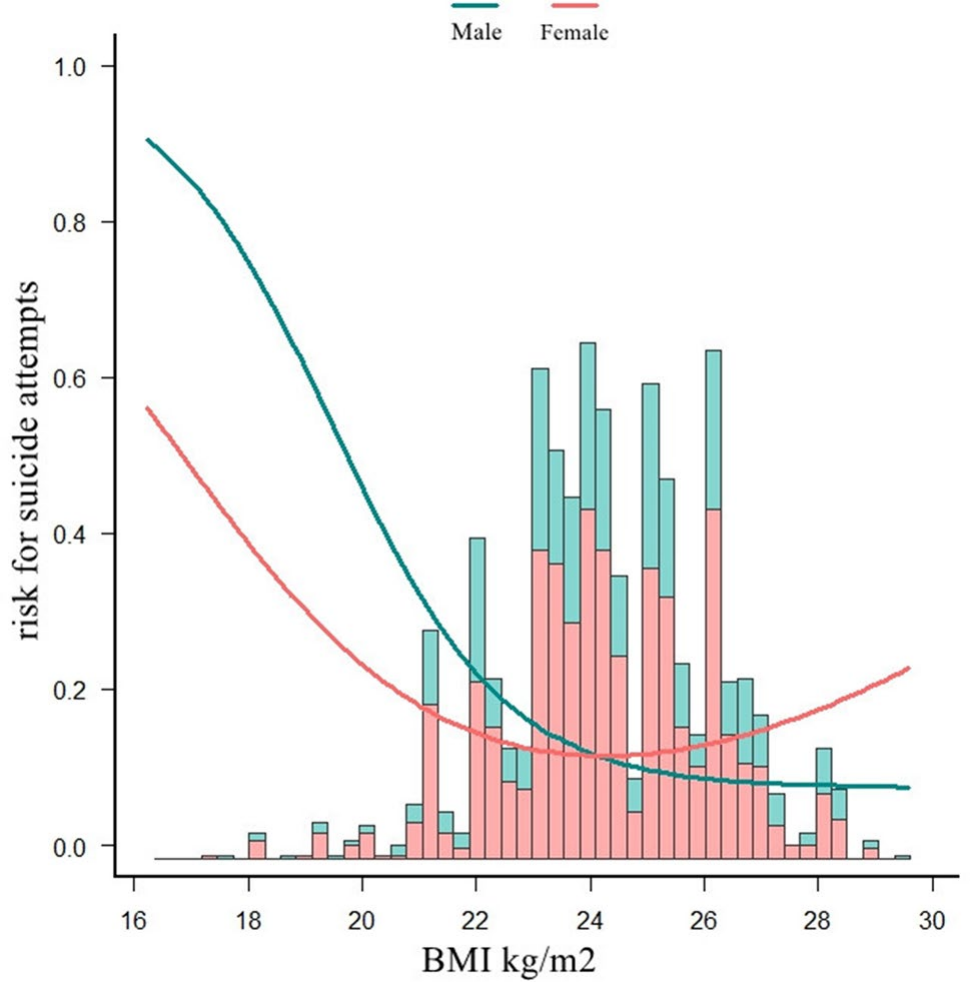
Association between BMI and SA in different gender of patients with FEDN MDD. A non-linear association between BMI and SA was found in the multivariable logistic regression model in both male and female patients (all *P* for non-linearity < 0.05). All adjusted for age at onset, duration of illness, education, HAMA, HAMD, TSH, TGAb, TPOAb, FBG, TC, TG, HDL-c, TG, LDL-c, systolic pressure, diastolic pressure.

An L-shaped relationship between SA and BMI was observed for both male and female patients by a piecewise regression model, and the estimated inflection point was 27.3 kg/m^2^ for males and 21.4 kg/m^2^ for females (Fig. 2 and Table 4). For men, once the BMI was lower than 27.3 kg/m^2^, a significantly negative association between BMI and SA was found (OR = 0.75, 95% CI 0.66–0.86, *P* < 0.001), while no significant BMI–SA association was detected (OR = 1.71, 95% CI 0.93–3.15, *P* = 0.087) if the BMI was higher than 27.3 kg/m^2^. In women, the risk of SA was negatively associated with BMI (OR = 0.48, 95% CI 0.32–0.72, *P* < 0.001) if the BMI was lower than 21.4 kg/m^2^, but no significant BMI–SA association (OR = 1.06, 95% CI 0.96–1.16, *P* = 0.255) was detected when the BMI was higher than 21.4 kg/m^2^. The dose–response relationship was agreed upon with the threshold effect analysis, and *P* was < 0.001 for the log-likelihood ratio test (Table 4) in both gender groups, indicating a non-linear association between BMI and SA.

**Table 4:** Threshold effect of body mass index and Suicide attempts using piecewise logistic regression model in different sex.

Inflection point of BMI (kg/m^2^)	OR	95% CI	*P*-value
Males			
< 27.3	0.75	0.66–0.86	< 0.001
> = 27.3	1.71	0.93–3.15	0.087
Log likelihood ratio test			< 0.001
Females			
< 21.4	0.48	0.32–0.72	< 0.001
> = 21.4	1.06	0.96–1.16	0.255
Log likelihood ratio test			< 0.001

All adjusted for age at onset, duration of illness, education, HAMA, HAMD, TSH, TGAb, TPOAb, FBG, TC, TG, HDL-c, LDL-c, systolic pressure, diastolic pressure.

### Exploration of modifier and interaction effects on the SA‑BMI association

**Figure 3:**
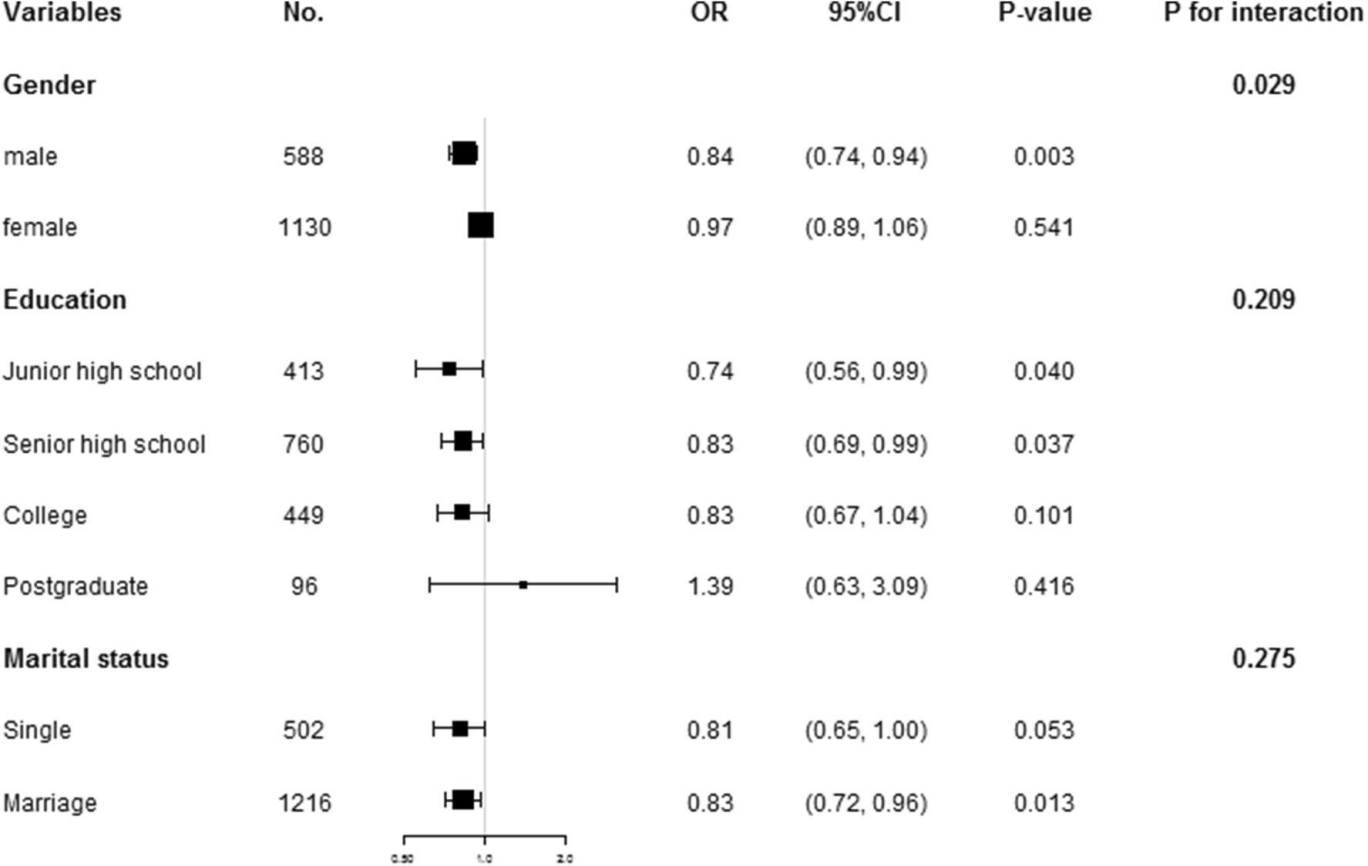
Stratified and interaction analyses of the association between BMI and SA. The OR (95% CI) was derived from the Logistic regression model. (Age at onset, duration of illness, HAMA, HAMD, TSH, TGAb, TPOAb, FBG, TC, TG, HDL-c, TG, LDL-c, SBP and DBP were adjusted).

We explored potential modifier or interaction effects from sex, education, and marital status, and found that only sex might be a potential interaction factor for the SA-BMI association (*P* for interaction = 0.029) (Fig. 3).

To elucidate the relationship between BMI and SA risk in specific demographic groups, male and female patients were stratified by age (< 20 years, 20–29 years, 30–39 years, > = 40 years) for subgroup analyses (Table 5). Among males, BMI exhibited a negative correlation with SA risk in the 20–29 years age group (OR = 0.51, 95% CI 0.35–0.74, *P* < 0.001), with a marginally significant interaction test (*P* for interaction = 0.013). For females, BMI also showed a negative correlation with SA risk, but specifically in the < 20 years age group (OR = 0.63, 95% CI 0.41–0.96, *P* = 0.033), and the interaction test was statistically significant (*P* for interaction = 0.071).

**Table 5:** Subgroup analysis in different age groups between BMI and SA.

	n	OR	95% CI	*P*-value
Males (years)				
< 20	98	1.13	0.87–1.48	0.353
20–29	172	0.51	0.35–0.74	< 0.001
30–39	130	0.85	0.61–1.19	0.348
> = 40	188	0.80	0.63–1.03	0.084
P for interaction				0.013
Females (years)				
< 20	131	0.63	0.41–0.96	0.033
20–29	288	1.03	0.83–1.26	0.805
30–39	237	1.05	0.84–1.31	0.688
> = 40	474	0.96	0.83–1.12	0.635
*P* for interaction				0.071

All adjusted for age at onset, duration of illness, education, HAMA, HAMD, TSH, TGAb, TPOAb, FBG, TC, TG, HDL-c, LDL-c, systolic pressure, diastolic pressure.

## Discussion

To our knowledge, this is the first study to examine the nonlinear association between BMI and SA in a relatively large sample of FEDN MDD patients, stratified by gender. Our study found that this relationship was significantly moderated by gender. A L-shaped nonlinear correlation between BMI and SA was observed in both genders, with a threshold effect at inflection points of 27.3 kg/m^2^ in males and 21.4 kg/m^2^ in females. BMI below the inflection point was negatively associated with SA in both genders, while no significant association was found above the inflection point.

Accumulating evidence suggests that underweight is a common comorbidity of SA, with the risk increasing as BMI decreases^27^. In a meta-analysis, Perera et al.^28^ found that among adult populations (> = 18 years old), underweight people had the highest risk of committing suicide compared to those of normal weight, but obesity and overweight people were substantially related to a lower risk of suicide. In large Swedish cohort research, Batty et al.^29^ showed an increased risk of SA in underweight men, and they also found that low weight was inversely correlated with SA within the normal BMI range. Individuals with a low BMI are predisposed to diminished physical functioning, which in turn is associated with emotional distress and more severe depressive symptoms^16^. A study conducted in the United States found that among men with MDD, a lower BMI was correlated with increased depressive symptom severity^30^. A recent systematic review by Amiri et al. explored the association between obesity and suicide over the past decade and showed an inverse association between overweight and obesity and SA and suicide mortality, while the association between overweight and obesity was positively associated with suicidal ideation^31^. Research by Carpenter et al.^32^ showed that a lower BMI or different weight status was associated with the probability of past-year SA and suicidal ideation in a sample of the general adult male population in the United States. This phenomenon, known as the obesity paradox, is supported by mounting evidence that a higher BMI may be protective in some patient populations^32^. Our findings provide credence to the obesity paradox in FEDN MDD patients. Samaan et al.^33^ found that the adiposity and obesity-associated (FTO) gene rs9939609 obesity-risk variant was paradoxically inversely associated with the risk of depression at the observational level, supporting the idea that the relationship between obesity and MDD can be explained, at least in part, by shared genetic factors. Adipose tissue is now recognized not only as an energy reservoir but also as a dynamic endocrine organ that secretes many adipocytokines with high metabolic activity. These cytokines are associated with metabolic anomalies and neurohormonal activity, including sympathetic nervous system activation, renin–angiotensin–aldosterone system activation, and leptin regulation^34^. Leptin levels may be linked to a higher risk of suicidal behavior, according to a recent meta-analysis^35^. Westling et al.^36^ reported that leptin levels in cerebrospinal fluid (CSF) were involved in neuroendocrine dysfunction in MDD patients who made SA. In addition to biological factors, current sociocultural influences on body image, particularly social media, further propagate an attentional focus on modifying specific aspects of one’s physique (e.g., enhancing muscularity, decreasing body fat, altering shape)^37^. This hyper-focused scrutiny of certain body regions can potentiate anxiety and distress surrounding particular features among adult women. It is hypothesized that such societal pressures, coupled with ones own motivations for thinness, may engender psychological distress in individuals at weight extremes compared to population norms^38^. Meanwhile, in this study, a similar L-shaped non-linear relationship between BMI and SA was determined for male or female patients, and there is no incremental advantage when BMI is greater than the threshold point.

However, evidence on the BMI–SA association is mixed. Some research has shown no association between BMI and SA^39^, while others have reported a positive association. Zhang et al.^40^ discovered that among 104,907 adolescents aged 12–15 years in 45 low- and middle-income countries (LMICs), being overweight and obese were significantly associated with a higher risk of SA. According to a recent review by Heneghan et al., Americans who are obese are more likely to commit suicide^41^. Individuals with bipolar disorder showed a similar association, with obese patients being almost twice as likely to have attempted suicide in the past as patients of normal weight^42^. These findings that are inconsistent with the results of this study may be due to the following factors: (1) Previous studies only focused on the linear relationship between SA and BMI, while our results examine the non-linear relationship for both men and women. (2) The participants in this study are special since the study population was made up of FEDN MDD patients, as opposed to the general community or individuals who had received depression treatment in many other studies. (3) The cultural and ethnic differences (e.g., lifestyle, beliefs, values, genetic factors, etc.) between the West and the East may have an impact on the prevalence of obesity, depression, and SA in the population^43^. For instance, the well-known Chinese proverb “laugh and gain fat” refers to the relationship between enjoyment and obesity. The Chinese consider that being overweight is a sign of good fortune because lucky people can eat more and put on more weight. However, discrimination is frequently associated with being overweight in Western nations. (4) The study population’s low average BMI of 24.37 ± 1.92 kg/m^2^ and the limited sample size of obese participants (BMI >  = 28 kg/m^2^, 64 in total) may have some bearing on the study’s findings.

Sex differences are observed in both clinical and epidemiological features of MDD. According to studies, women are more likely than men to experience MDD^44^. Females with MDD are more likely to exhibit increased anxiety, along with higher rates of comorbid anxiety disorders, whereas males with MDD are more likely to show increased psychomotor agitation and to have comorbid substance abuse disorders^45^. Males with MDD are more likely to succeed in their attempts at suicide than females, despite the fact that females with MDD are more likely to attempt suicide^46^. This study found that although there was an L-shaped nonlinear correlation between BMI and SA in both male and female patients with depression, there were differences in inflection points, with 27.3 kg/m^2^ in males and 21.4 kg/m^2^ in females, which has not been reported in previous studies. Although there were gender differences in the relationship between BMI and SA in patients with FEDN MDD, the mechanisms behind these observations remain unclear, and several studies indicate that differences in sex hormone levels may partially explain the effects. Given that depressive disorders and autoimmune diseases are more common in females, sex hormones appear to be a likely factor in sex differences in cytokine-associated depression^47^. It is possible that sex-specific hormonal substrates contributed to the observed sex differences in cytokine-associated depressed mood at the neurochemical level^48^. Estrogen therapy shows efficacy for perimenopausal depression, supporting interactions between serotonin and gonadal hormones underlying sex differences in the antidepressant response^49–51^. Additionally, estrogen has also been proposed to potentially help explain sex-related differences in depression. Nevertheless, there is evidence to suggest that estrogen may influence mood through regulating various aspects of noradrenergic, serotonergic, GABAergic, and dopaminergic transmission^52^. The sexually dimorphic features in the cortico-limbic-striatal neural system may correspond to the sex differences in clinical observations of MDD and are associated with mood and emotion regulation dysfunction in MDD^53^. Sex-related patterns of abnormalities within the cortico-limbic-striatal nervous system, such as predominant prefrontal-limbic abnormalities in MDD females versus predominant prefrontal-striatal abnormalities in MDD males, suggest that differences in neural circuitry may mediate gender differences in the clinical presentation of MDD and potential targets for gender-differentiated treatment of the disorder^54^. Finally, because of the different contexts in which men and women develop as children and adolescents, there may be additional cognitive and psychological elements that influence gender disparities^52^. During prenatal and adolescent development, sex hormones may influence a number of behaviors, including psychological changes during puberty (e.g., increased risk-taking behavior in early adolescence, gender differences in internalizing symptoms, i.e., girls have more anxiety and self-esteem problems than boys), gender-typed activity interests (e.g., career choices), gender identity, sexual orientation, cognitive abilities, and behavioral problems^55^. Previous research on cognitive development and the effects of gender coping styles has shown differences in boys’ and girls’ preferences for coping strategies^56^. In addition, a longitudinal study of depressive cognitions in children and adolescents found that some cognitive differences in girls, including perceptions of appearance and self-worth, were more stable and idiosyncratic than those of boys before gender differences in depression emerged^57^. Female patients in this study had a higher age and age of disease onset than males, which may have influenced the relationship between SA and BMI to some extent, although we have controlled for this in the multifactorial regression model. In the future, it will be necessary to match relevant factors, such as age, to further validate the results of this study.

This study has several limitations. First, patients were recruited from one hospital in Taiyuan, China, and were ethnically Han Chinese. Confirmation in diverse populations is needed. Second, the cross-sectional design cannot determine causal relationships between BMI and SA. Future longitudinal studies should investigate this causal association. Third, SA were identified using interviews and medical records rather than structured diagnostics, without details on severity or ideation. Specific suicide assessments are needed in future work. Fourth, the participants in this study were MDD patients in their first episode who had not received any medication for their depressive symptoms. Even though there was a benefit to selecting first-episode and drug-naïve patients, we cannot entirely exclude patients whose diagnosis may have changed to bipolar disorder since the original depressed episode may have paralleled the MDD episode. Finally, unmeasured confounders like smoking, alcohol use, personality, income, social factors, and biological variables could influence the BMI-suicide attempt relationship. Future research should account for additional confounders to elucidate the underlying mechanisms.

## Conclusions

In conclusion, this study showed an L-shaped nonlinear association between BMI and SA in Chinese FEDN MDD patients for both men and women, with inflection points of 27.3 kg/m^2^ for men and 21.4 kg/m^2^ for women, respectively. The findings provided academics with an exciting psychopathology viewpoint on how to reduce suicide risk in FEDN MDD patients and prevent suicide. Furthermore, because of the study’s limitations, such as its cross-sectional design, absence of a structured evaluation instrument, and inability to collect some relevant data, the findings of this investigation should be regarded as preliminary and verified in a follow-up study employing a longitudinal design.

## Data Availability

The datasets used and/or analysed during the current study available from the corresponding author on reasonable request.
